# Regulation of antibiotic persistence and pathogenesis in *Acinetobacter baumannii* by glutamate and histidine metabolic pathways

**DOI:** 10.1186/s12866-024-03654-1

**Published:** 2025-02-14

**Authors:** Ho Seok Sim, Yong-Kook Kwon, Hokyung Song, Geum-Sook Hwang, Jinki Yeom

**Affiliations:** 1 Department of Biomedical Science, College of Medicine, Seoul National University, Seoul, 03080 Republic of Korea; 2 Integrated Metabolomics Research Group, Western Seoul Center, Korea Basic Science Institute, Seoul, 03760 Republic of Korea; 3 Division of Food Safety Risk Assessment, National Institute of Food and Drug Safety Evaluation, Cheongju, 28159 Republic of Korea; 4 Graduate School of Analytical Science and Technology, Chungnam National University, Daejeon, 305-764 Republic of Korea; 5 Department of Environmental Engineering, Chosun University, Gwangju, 61452 Republic of Korea; 6 College of Pharmacy, Chung-Ang University, Seoul, 06974 Republic of Korea; 7 Department of Microbiology and Immunology, College of Medicine, Seoul National University, Seoul, 03080 Republic of Korea; 8 Cancer Research Institute, Seoul National University, Seoul, 08826 Republic of Korea

**Keywords:** *Acinetobacter baumannii*, Antibiotic persistence, Pathogenesis, Metabolomics, Metabolic pathways

## Abstract

**Background:**

Metabolite production is essential for the proliferation and environmental adaptation of all living organisms. In pathogenic bacteria, metabolite exchange during host infection can regulate their physiology and virulence. However, there is still much unknown about which specific metabolic pathways in pathogenic bacteria respond to changes in the environment during infections. This study examines how pathogenic bacterium *Acinetobacter baumannii* uses particular metabolic pathways to regulate its ability to antibiotic persistence and pathogenesis.

**Results:**

To determine specific metabolic pathways in pathogenic antibiotic resistance bacteria, metabolite profiles of bacteria were constructed using ultraperformance liquid chromatography/quadrupole time-of-flight mass spectrometry and multivariate statistical analysis. *A. baumannii* generates amino acid derivative metabolites, which are precursors for fatty acid production. Comparative genomic analysis identified specific genes regulating the production of these metabolites and fatty acids in *A. baumannii*. Inactivation of genes involved in glutamate metabolism, *gdhA*, *aspB*, *murI1*, and *racD*, impairs antibiotic persistence, while inactivation of the *hisC* gene, encoding histidinol − phosphate aminotransferase enzyme in histidine metabolic pathway, increases bacterial survival inside macrophages during infections.

**Conclusions:**

This study reports that *A. baumannii* regulates antibiotic persistence and pathogenesis through glutamate and histidine metabolic pathways, respectively. These findings suggest that specific metabolic pathways regulate bacterial pathogenesis and antibiotic persistence during infections, providing potential therapeutic targets for pathogenic bacteria.

**Supplementary Information:**

The online version contains supplementary material available at 10.1186/s12866-024-03654-1.

## Introduction

All living cells universally respond to environmental stresses by adjusting the synthesis of RNA, protein, and metabolites to ensure growth and survival. As end products, metabolites play a crucial role in regulating cellular functions both intracellularly and intercellularly. When confronted with challenges such as nutrient disturbances during infections, bacteria adapt their metabolic processes, resulting in the production of diverse metabolites [1–7]. These metabolites also regulate interactions both among bacteria themselves and between bacteria and host cells [1, 4, 8, 9]. However, little is known about how bacterial species modulate metabolic pathways to survive under antibiotic treatment during infections.

When faced with adverse conditions, such as nutrient deprivation, antibiotic exposure, or immune-mediated challenges, bacteria can enter a metabolically dormant state [10]. This phenomenon, known as antibiotic persistence or tolerance, allows these quiescent cells to evade antibiotic lethality by temporarily inactivating the biological pathways targeted by the antibiotics [11]. In clinical settings, persister bacteria cause the relapse of infections [12], as persister bacteria, unlike tolerant bacteria, can revert to an active metabolic state upon encountering favorable conditions [13]. Thus, this study will focus on antibiotic persistence, not tolerance, under antibiotic treatment. Furthermore, persister bacteria have been observed to secrete indole metabolites, which may promote the dissemination of antibiotic persistence within bacterial populations [5]. Despite these findings for several decades, the specific metabolomic and genomic adaptations underlying antibiotic persistence remain poorly understood. To bridge this knowledge gap, we conducted a comprehensive analysis of the bacterial metabolome and genome to investigate the molecular mechanisms governing antibiotic persistence.

*Acinetobacter baumannii*, a ubiquitous opportunistic pathogen, is frequently associated with hospital-acquired infections [14]. The World Health Organization (WHO) has designated multidrug-resistant *A. baumannii* as a critical priority pathogen, emphasizing the pressing need for innovative therapeutic approaches [15]. Although various antibiotic resistance mechanisms, such as antibiotic degradation and target modifications, are well-established [16, 17], the means by which *A. baumannii* regulates its metabolic pathways under antibiotic treatment to maintain persistence remain elusive. Indeed, *A. baumannii* causes nosocomial infections through its ability to survive on surfaces [18] which contributes to fatal sepsis in many patients [19]. However, little is known about how it survives inside host innate immune cells during infections. In this study, we conducted a thorough metabolic analysis to identify specific metabolites that play a crucial role in regulating antibiotic persistence and pathogenesis in *A. baumannii*.

In this study, we found that the representative antibiotic resistant bacterium *A. baumannii* generates unique metabolites pivotal for antibiotic persistence and virulence. Our comprehensive metabolite profiling reveals that *A. baumannii* notably changes certain metabolic pathways, particularly those associated with amino acid derivatives and fatty acids. Using comparative genomics and physiological assays, we identified specific genes associated with glutamate metabolism play a crucial role in antibiotic persistence. Furthermore, inactivation of histidine biosynthetic pathways increases bacterial survival inside host innate immune cells. These metabolic pathways will be a potential target for the development of new antibiotics, as it provides novel insights into antibiotic persistence in pathogenic bacteria.

## Results

### *A. Baumannii* exhibits specialized metabolite production

Metabolites represent the products of genetic processes in living beings that regulates cellular physiology. Bacteria generate unique metabolites that govern various physiological activities within them. For instance, *Escherichia coli* utilizes the indole metabolite to enhance antibiotic persistence and dispersion within their species [5]. Moreover, previous studies have revealed how metabolic pathways are regulated in various bacteria [20]. However, there is limited knowledge about which metabolic pathways are preferred to regulate physiology in different bacterial species. Thus, we conducted a comprehensive metabolite profiling study to pinpoint specific metabolites in bacterial species.

To investigate the bacterial metabolome, we chose four Gram-negative bacterial strains for our study, as detailed in Supplementary Table 1. These strains include the commonly studied *E. coli* K-12, its pathogenic relative *E. coli* O157:H7, and two multi-drug resistant pathogens, *Pseudomonas aeruginosa* PAO1 and *Acinetobacter baumannii* ATCC 17978.

**Fig. 1:**
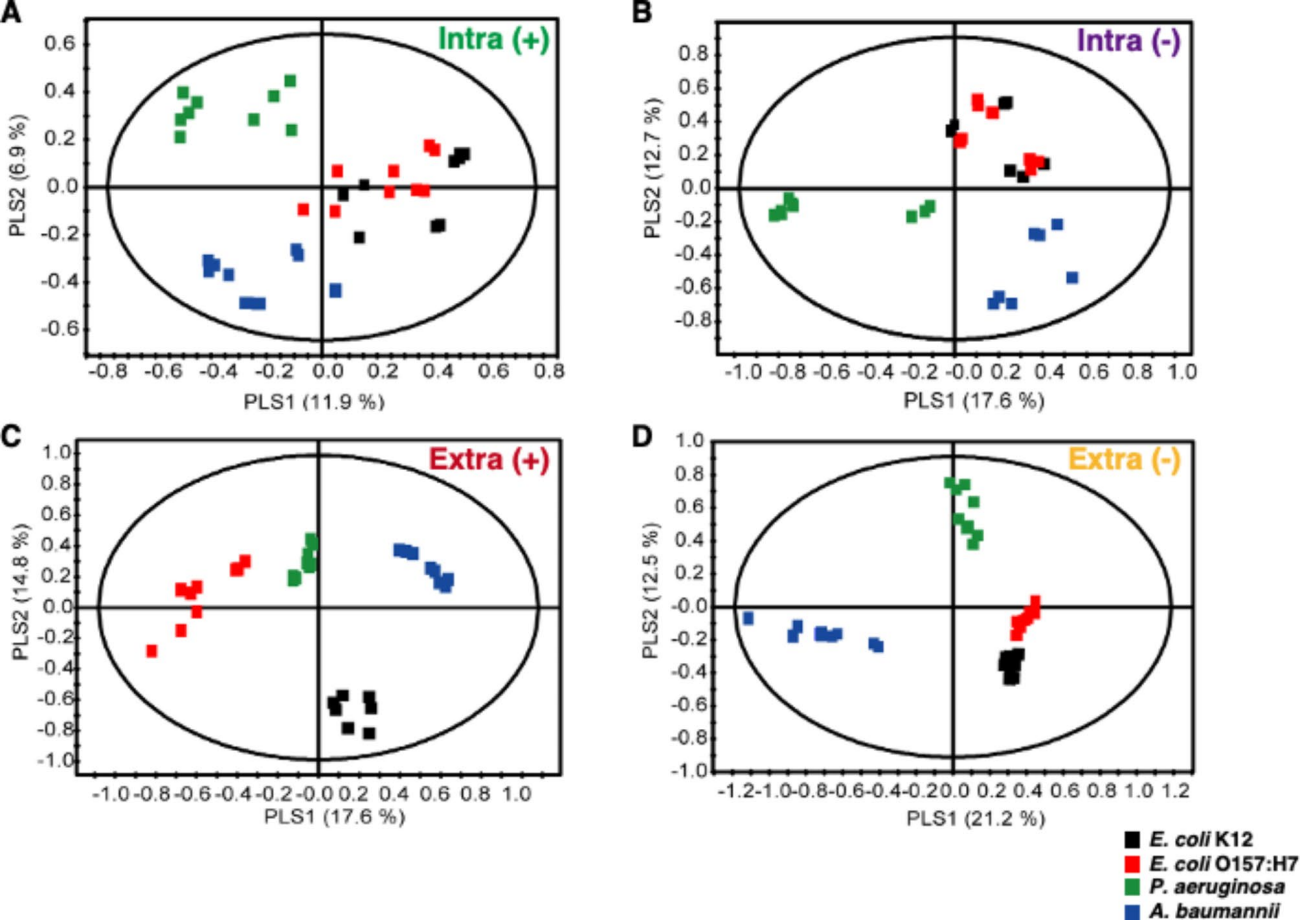
PLS-DA score plots derived from bacterial samples. PLS-DA score plots of bacterial samples demonstrated clear metabolic differences. Intra(+), intra-metabolite assessed by ESI-positive mode (**A**); Intra(-), intra-metabolite assessed by ESI-negative mode (**B**); Extra(+), extra-metabolite assessed by ESI-positive mode (**C**); Extra(-), extra-metabolite assessed by ESI-negative mode (**D**)

We performed a multivariate statistical analysis, Partial Least Squares Discriminant Analysis (PLS-DA), on the global metabolites found in bacteria (Fig. 1). To ensure the reliability of PLS-DA model, we conducted a permutation procedure using the equal number of components (Fig. S1). The analyses indicate that all four models are statistically robust (Fig S1 and Table S2). Interestingly, *P. aeruginosa* and *A. baumannii* are well-separated from other bacteria in both intra-metabolome and extra-metabolome negative modes, as they are located in different quadrants from other bacteria in the PLS-DA model (Fig. 1). In the extra-metabolome positive mode, metabolites from all species are well-separated (Fig. 1). Notably, these models demonstrate high robustness, with R_2_Y and Q_2_ values closer to 1 (Table S2). A detailed summary of the patterns observed during the multivariate statistical analysis is provided in Supplementary Table 2.

Based on the PLS-DA model, the *A. baumannii* samples displayed distinct metabolite patterns compared to other samples. This is evident from the fact that the metabolome of *A. baumannii* consistently occupied a different quadrant in all PLS-DA plots (Fig. 1), while other bacterial samples shared the same quadrant in at least one of the PLS-DA plots. This suggests that *A. baumannii* possesses a unique global metabolome pattern. Given that, we hypothesized that certain metabolites might play a role in regulating specific phenotypes within *A. baumannii*.

### *A. Baumannii* specifically produces amino acid derivatives and lipid metabolites

**Fig. 2:**
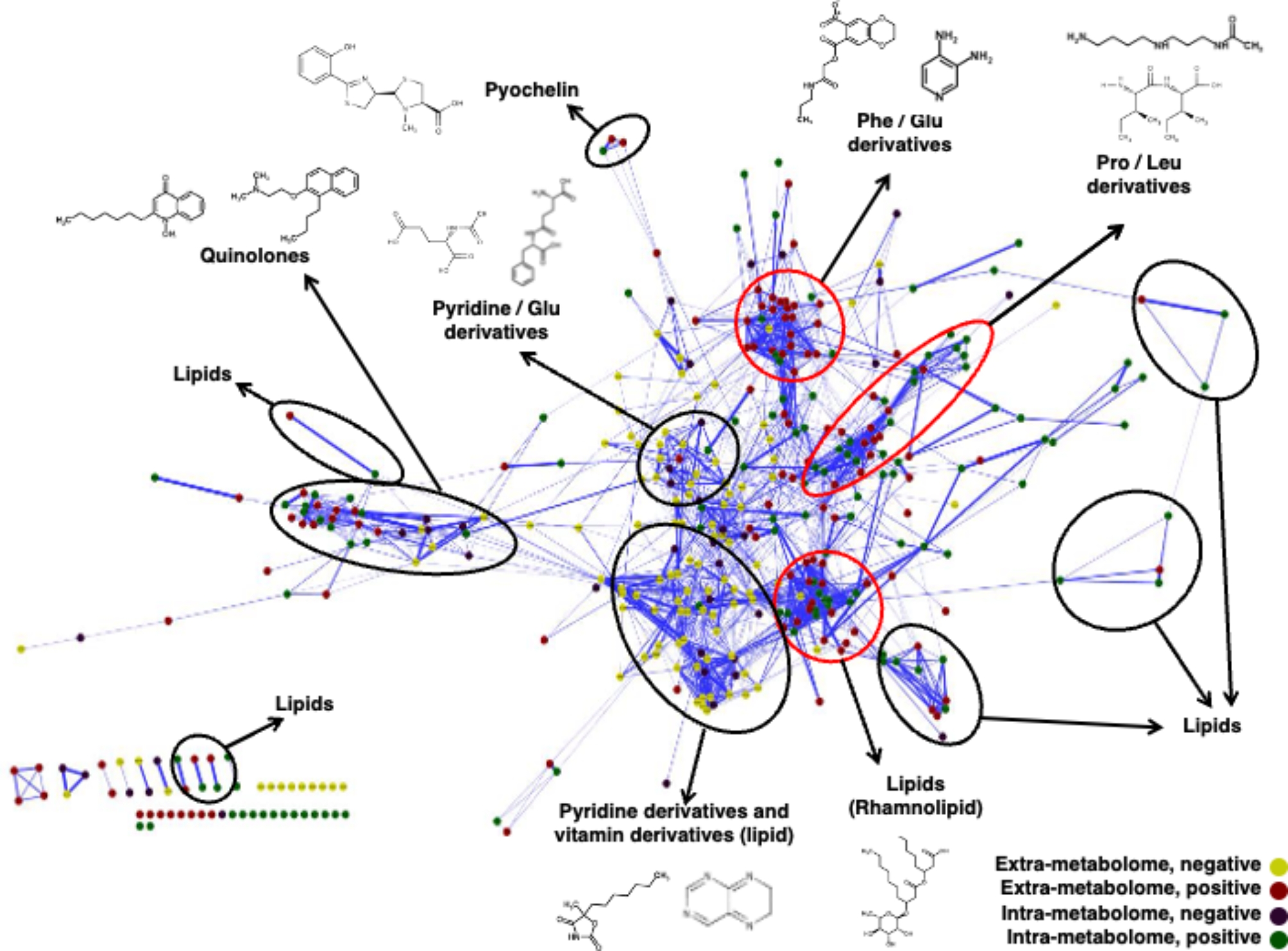
Metabolite networks of MS/MS fragmentation data obtained from bacterial metabolomes. Metabolite networks were generated using the Cytoscape program with cosine vales of bacteria metabolome. The structures of each of the annotated clusters are shown in Supplementary Dataset 1. The node color indicates LC/MS analytic modes: yellow nodes represent extra-metabolites assessed by ESI-negative mode; red nodes represent extra-metabolites assessed by ESI-positive mode; purple nodes represent intra-metabolites assessed by ESI-positive mode; green nodes represent intra-metabolites assessed by ESI-positive mode. The thickness of a link between two nodes denotes the likeness score for that pair of spectra; thus, pairs with higher scores are represented by thicker lines and are usually placed closer together. Circle indicated that metabolites have similar chemical structures and are likely part of the same metabolic pathways. Specific metabolites from *A. baumannii* are highlighted with red circles

**Fig. 3:**
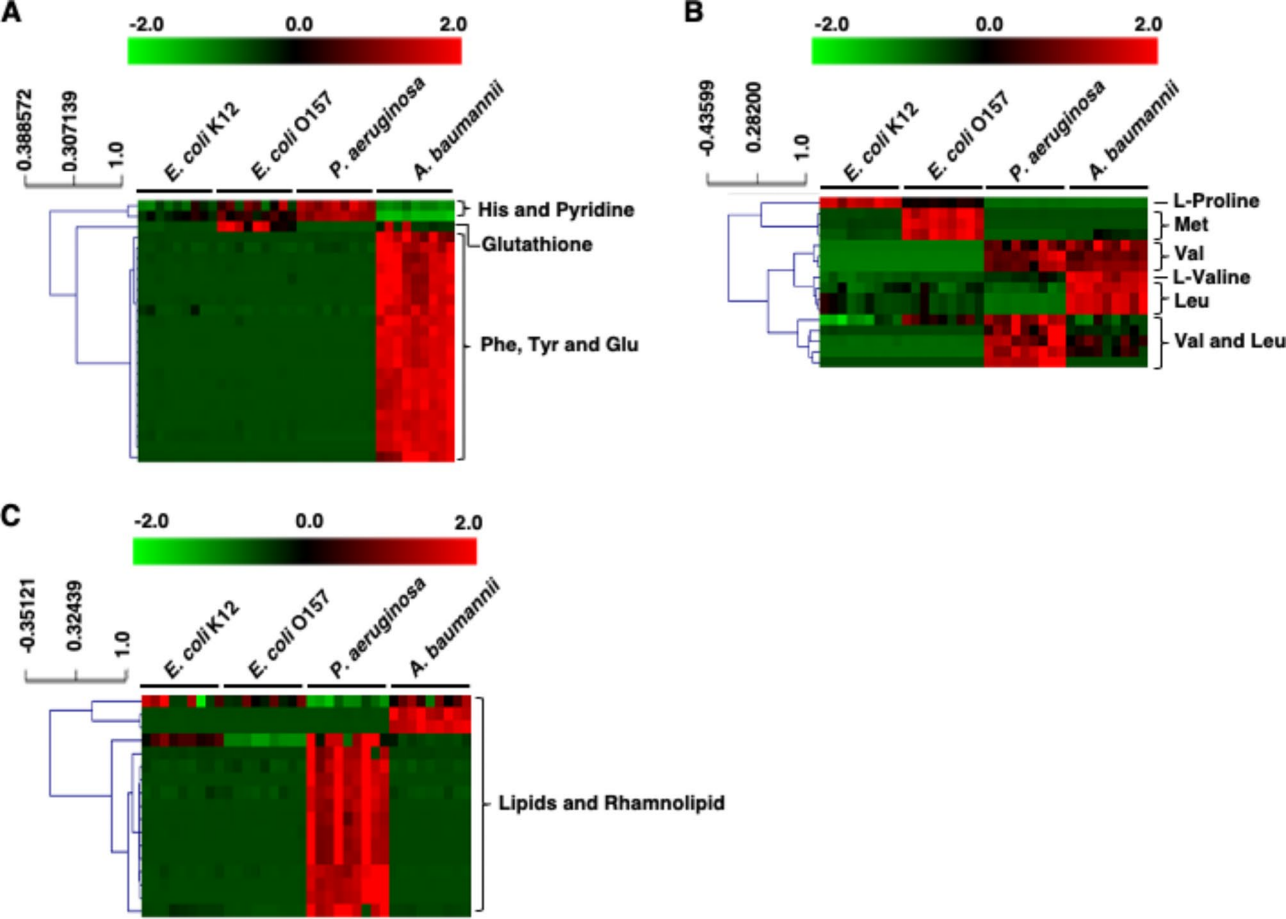
Partial annotated Heat map and HCA analysis depicting compounds of the MS/MS network. Heat map and hierarchical cluster analysis (HCA) analysis depicting compounds of the MS/MS network from Fig. 2 for Phe/Glu derivatives metabolites (**A**), Phe/Leu derivatives (**B**) and lipids metabolites (**C**). Name of metabolites such as Phe, Tyr, Glu, Met, Val, Leu refer to derivative metabolites of amino acids. Each row represents a metabolite, which corresponds to a node in Fig. 2 and each node indicates a different metabolite due to varying m/z values and MS spectrum. After LC-Q-TOF-MS analysis, all data were processed using MZmine software and the METLIN database to identify metabolite structures and names. The database suggested either exact names or derivatives of metabolites. Amino acid name such as Val and Leu indicate that these metabolites are produced with valine and leucine as backbones, but it is challenging to determine which is the primary backbone for producing Val and Leu derivative metabolites

To explore the unique metabolic pathways in *A. baumannii*, we depicted the metabolite connections using MS/MS fragmentation data from metabolites chosen through multivariate statistical analysis. We selected variables with a VIP value of over 1.5 in the PLS-DA model, and each of these was deemed significant in the false discovery rate (FDR) test (Dataset S1). These factors were then employed to form molecular networks (Fig. 2). These networks were illustrated where each node (i.e., circle) signifies a unique consensus MS/MS spectrum related to a specific parent mass. In addition, we conducted a hierarchical cluster analysis (HCA) to categorize metabolites based on notable variations in their relative abundance (Fig. 3). A list of notable metabolites in bacteria can be found in Table 1. We also indicated the MS/MS spectra for specific compounds (Fig. S3-7).

**Table 1:** Significant metabolites and pathways with MS/MS network and HCA

Strains	Identified metabolites
*E. coli* K-12	Proline, Carnitine and Lipid metabolism, Vitamine metabolism, Pyridine and Glu derivatives
*E. coli* O157:H7	Glutathione, Met derivatives, Pyridine derivatives, Lipids
*P. aeruginosa* PAO1	Quinolines, His and Pyridine derivatives, Val and Leu derivatives, Rhamnolipid, Branched fatty acid, Pyochelin, Glu derivatives
*A. baumannii 17978*	Phe and Tyr derivatives, Leu and Val derivatives

**Fig. 4:**
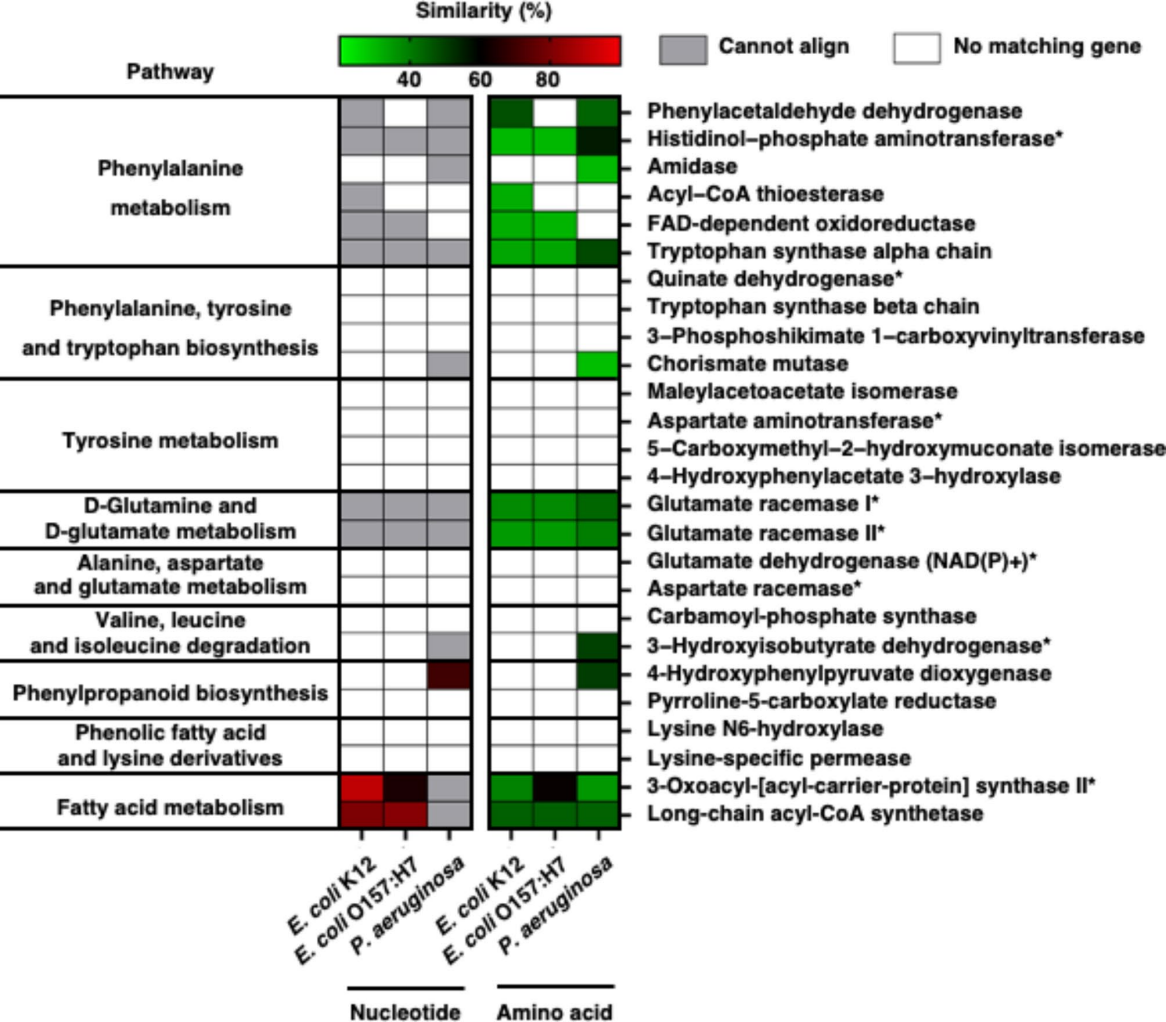
Comparison of genes and proteins similarity of *A. baumannii* with other bacteria. Graph indicates the similarity of *A. baumannii* nucleotide and amino acid sequence between *E. coli* K-12, *E. coli* O157:H7 and *P. aeruginosa*. Similarity was analysis with BLASTn and BALSTp in NCBI database. (Cannot align: gene was found in bacterial genomic database but cannot align to analysis. No matching gene: gene was not found in bacterial genomic database)

**Fig. 5:**
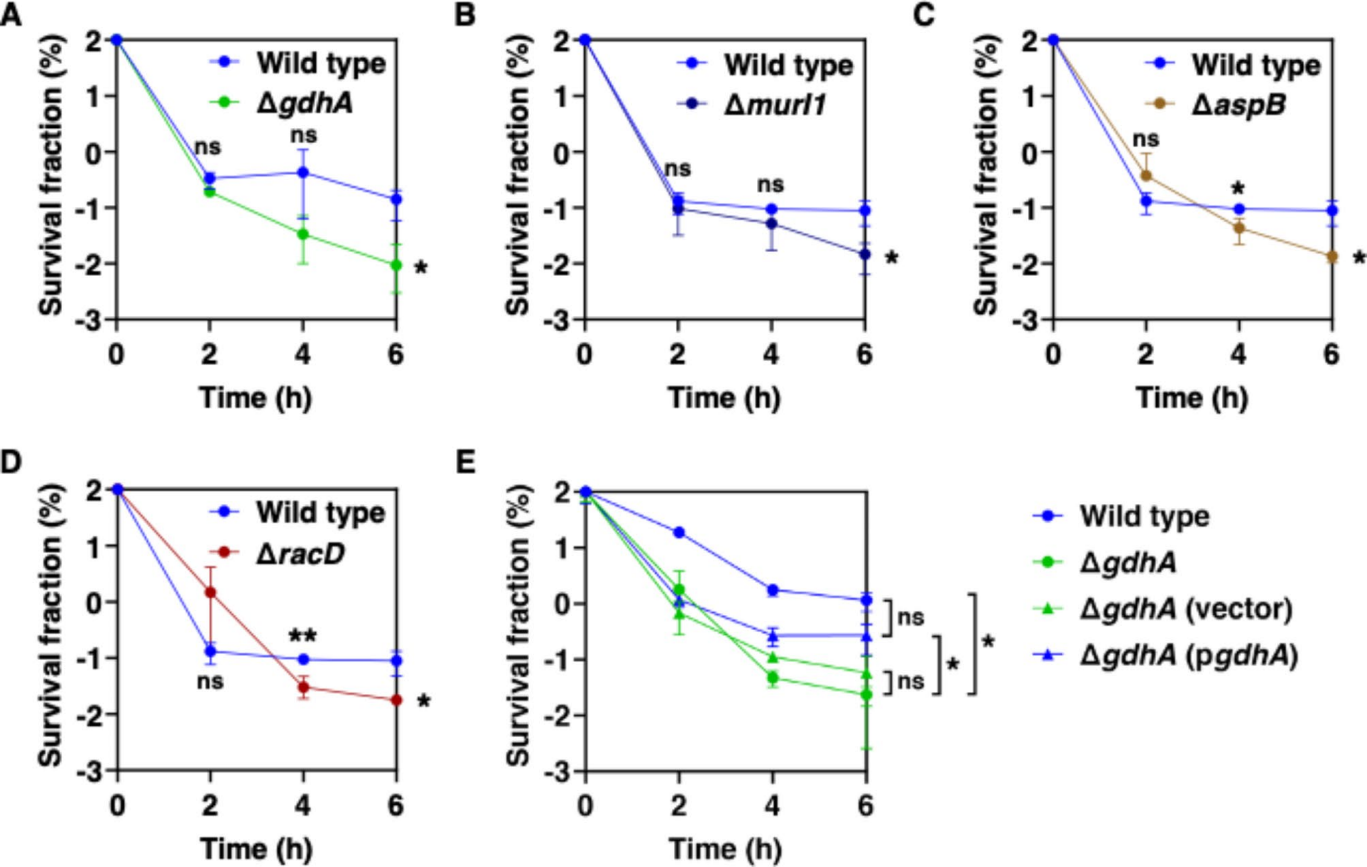
Time killing curve of *A. baumannii* under antibiotic treatment. Time Killing curves of wild type and Δ*gdhA*, Δ*murI1*, Δ*aspB* and Δ*racD* (**A-D**) and *gdhA* complementation (**E**). Norfloxacin (100 µg/ml) was treated at early stationary phase bacteria following growth in M9 media and samples were collected every 2 h after treatment. CFU was measured after removing the antibiotics by washing with PBS. Survival fraction was calculated with bacterial number before antibiotic treatment. Statistical analyses were performed using GraphPad Prism software. Unpaired Student’s t tests were performed on the wild-type sample and the other combinations (*p < 0.05, **p < *0.01* and ns: no significant)

**Fig. 6:**
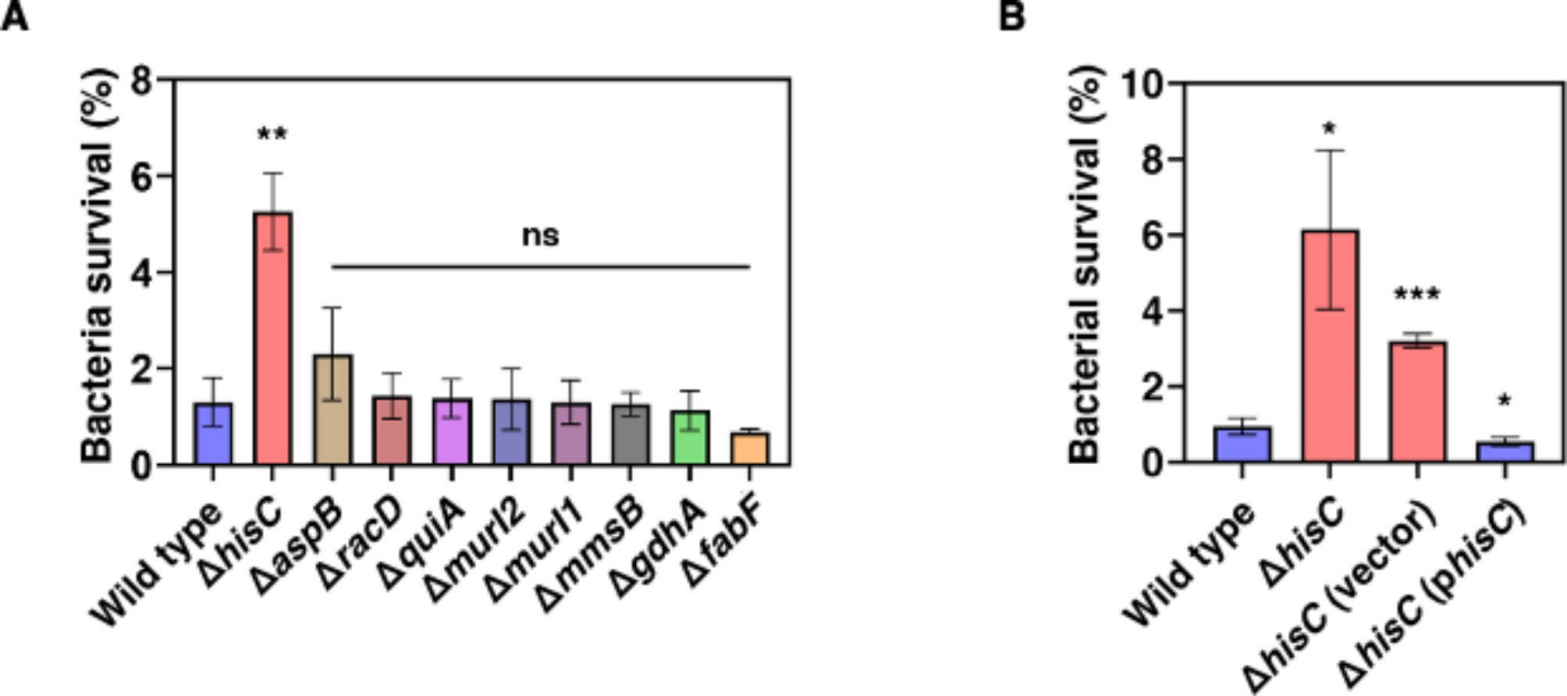
Intramacrophage survival rate of *A. baumannii*. (**A**) Intramacrophage survival rate of wild type and mutant strains and (**B**) Δ*hisC* complementation. Early stationary phase of wild type and mutant strains following growth in M9 media were infected with murine-derived macrophage J774A.1. Intramacrophage survival fraction was measured at 6 h.p.i. Survival rate was calculated depending on number of bacteria at 0 h.p.i. Statistical analyses were performed using GraphPad Prism software. Unpaired Student’s t tests were performed on the wild-type sample and the other combinations (*p < 0.05, **p < 0.01 and ***p < *0.001*)

**Fig. 7:**
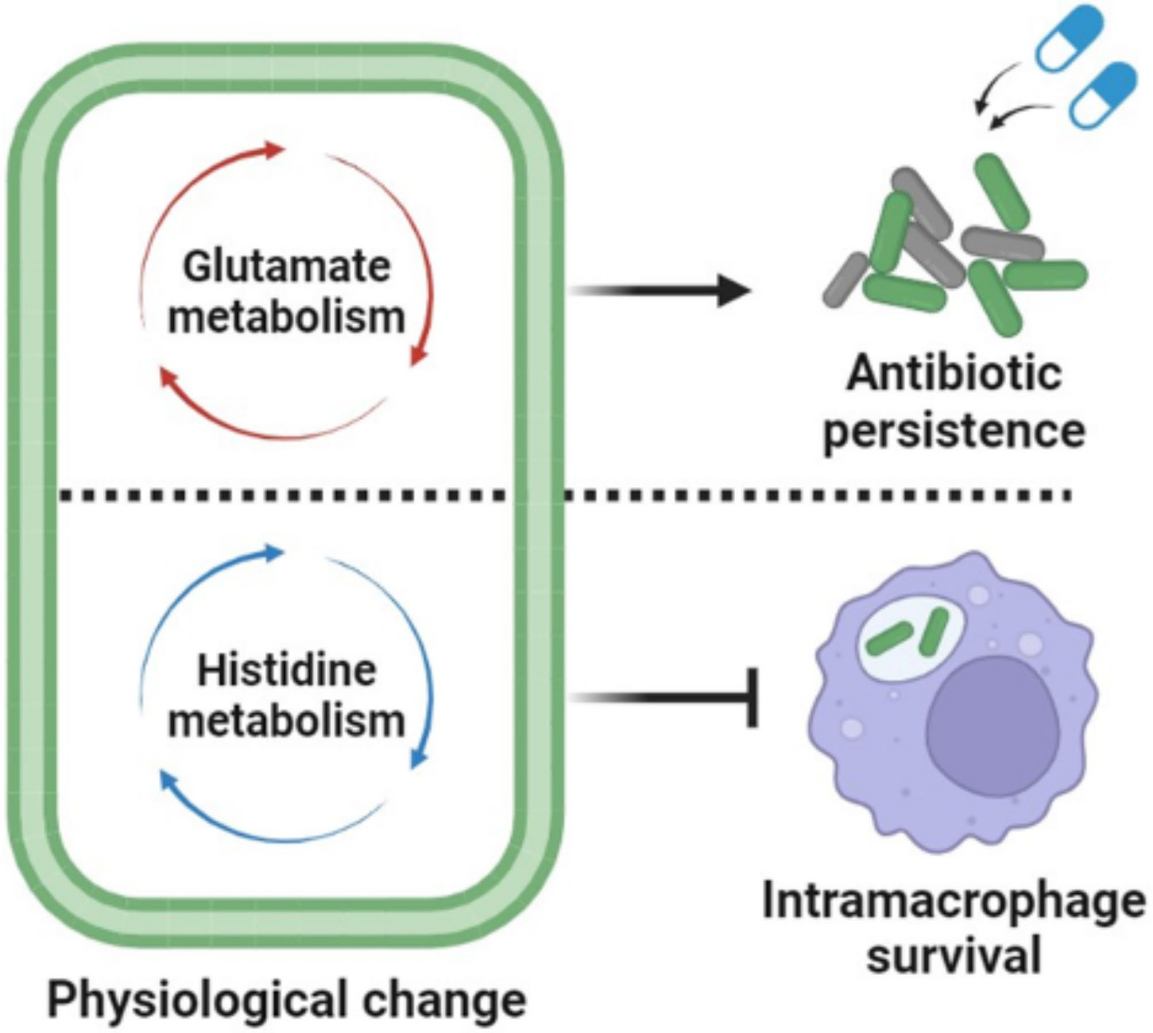
Integrated metabolomic-genomic analysis revealed species-specific physiological changes. Integrated metabolomic-genomic analysis revealed that glutamate metabolism is important in antibiotic survival in *A. baumannii*. Also, histidine metabolism inhibits bacterial survival inside macrophage

Interestingly, the metabolite profiles of *A. baumannii* were notably different from other bacteria when analyzed through molecular networking and HCA (See Fig. 2, highlighted by the red circle and Fig. 3). In particular, both extracellular and intracellular metabolites were significantly accumulated in *A. baumannii*, as detected in the positive ESI mode (Fig. 2, marked by the red circle). First, metabolites like phenylalanine and glutamic acid derivatives were exclusive to *A. baumannii* (Fig. 2). In detail, *A. baumannii* generates metabolites such as phenylalanine, tyrosine, and glutamic acid derivatives (Fig. 3A and S2), which can serve as the building blocks for various aromatic signaling molecules in bacteria [21]. Second, metabolites derived from proline and leucine were highly accumulated in *A. baumannii* (Fig. 2). Both leucine and valine, found in abundance in *A. baumannii* (Fig. 3B and S3), are hydrophobic amino acids that contribute to the structure and stability of hydrophobic proteins, including transporters and channels [22]. Additionally, these amino acids can act as an energy source for bacterial cellular functions [23]. And third, the presence of rhamnolipid and various lipids was noted in both *P. aeruginosa* and *A. baumannii* metabolites (Figs. 2 and 3C and S4). Rhamnolipids, primarily produced by *P. aeruginosa*, act as natural surfactants, emulsifiers, fungicides, and antibiotics in nature [24, 25]. *A. baumannii* can generate specific lipid metabolites including 3-O-alpha-L-rhamnopyranosyl-3-hydroxydecanoyl-3-hydroxydecanoic acid and its derivates (Fig. 3C and S4), which might play a role in regulating specific phenotypes within *A. baumannii*. In summary, *A. baumannii* distinctly produces specific metabolites such as amino acid derivative and lipids.

### *A. Baumannii* possesses distinct genes responsible for the synthesis of amino acid derivative metabolites and lipids

To identify the specific genes responsible for the unique metabolite profile of *A. baumannii*, we performed a comparative genomic analysis (Fig. 4 and Table S3). We first selected potential genes or proteins from the KEGG database that could generate the unique metabolites found in *A. baumannii*. Next, they compared these genes and proteins to those present in the genomes of *E. coli* K-12, *E. coli* O157:H7, and *P. aeruginosa* using nucleotide and protein BLAST (Table S3). If BLAST did not find any matching genes or proteins, it was indicated as “No matching gene.” Additionally, if the similarity scores were less than 60% for nucleotide BLAST and 30% for protein BLAST, they were labeled as “Cannot align.”

Intriguingly, many of the enzymes in *A. baumannii* that contribute to the production of specific metabolites after metabolome analysis are not found in other bacterial genomes (Fig. 4, No matching gene). Initially, nucleotide BLAST analysis revealed different identity of genes related to the metabolism and synthesis pathways of amino acids like phenylalanine, tyrosine, tryptophan, glutamic acid, valine, and leucine in other bacteria (Fig. 4). Additionally, genes encoding enzymes for fatty acid synthesis and metabolism were scarcely present in other bacterial genomes, with the exception of fatty acid metabolism genes in *E. coli* species (Fig. 4). Furthermore, while several enzymes associated to phenylalanine, glutamate, and fatty acid metabolism were detected at the protein level in other bacteria, their similarity to those in *A. baumannii* was very low (Fig. 4, green color), suggesting they might function differently. Taken together, *A. baumanni*i possesses distinctive gene and protein sequences when compared with the other bacteria that might contribute to differential metabolite profile (Fig. 4 and Table S3).

### Glutamate and histidine metabolic pathways regulate antibiotic persistence and bacteria survival inside macrophage, respectively, in *A. Baumannii*

Metabolic alterations have the potential to modulate various aspects of bacterial physiology, including survival within innate immune cells and antibiotic persistence [5, 26]. To determine whether specific metabolites play a role in regulating *A. baumannii* pathogenesis and antibiotic persistence, we generated mutant strains for representative enzymes from each metabolic pathway identified through comparative genome analysis (Fig. 4, asterisk mark). Our comparative genomic analysis revealed that nine specific metabolic pathways may be unique to *A. baumannii*, suggesting their potential importance in various physiological processes. Thus, we constructed single-gene deletion mutant strains for each pathway. Notably, the inactivation of these genes did not affect bacterial growth in M9 minimal medium (Fig. S8).

Multiple evidence supports the notion that glutamate metabolism regulates antibiotic persistence in *A. baumannii*. First, the inactivation of genes crucial to glutamate metabolism, such as glutamate dehydrogenase (*gdhA*), glutamate racemase (*murI1*), aspartate aminotransferase (*aspB*), and aspartate racemase (*racD*), was found to significantly reduce bacterial survival rates 6 h after 100 µg/ml norfloxacin treatment, which is the quinolone antibiotic targeting bacterial DNA gyrase [27] without changing MIC (16 µg/ml) (Fig. 5A-D). Moreover, we tested persistence assay against colistin (lipid membrane disruption), gentamicin (protein synthesis inhibition) and ciprofloxacin (DNA gyrase inhibition) to understand whether this phenomenon is norfloxacin or DNA gyrase targeting antibiotics specific or not. Colistin and ciprofloxacin treatments showed significantly decreased persistence at Δ*gdhA* while under gentamicin one it didn’t change (Fig S10). This result indicates that decrease of persistence is not a norfloxacin specific phenomenon. This highlights the essential role of these genes in antibiotic persistence of *A. baumannii*. By contrast, the mutation of genes involved in other metabolic pathways, including 3-hydroxyisobutyrate dehydrogenase (*mmsB*), 3-Oxoacyl-[acyl-carrier-protein] synthase II (*fabF*), histidinol − phosphate aminotransferase (*hisC*), quinate dehydrogenase (*quiA*), and another isoform of glutamate racemase (*murI2*), did not show a significant effect on antibiotic persistence under norfloxacin treatment compared to wild-type strain (Fig. S9). Second, bacterial survival is considerably higher in the *gdhA* mutant carrying a multi-copy plasmid with the *gdhA* gene expressed from a heterologous promoter compared to an isogenic strain with the plasmid vector control (Fig. 5E).

This study examines how pathogenic bacterium *A. baumannii* uses particular metabolic pathways to regulate antibiotic persistence and pathogenesis. The inactivation of *hisC* led to a significant increase in the presence of bacteria inside macrophages by approximately four-fold 6 h post-infection, compared to the wild type (Fig. 6A). The observed bacterial survival inside macrophages was attributed to the absence of *hisC*, as evidenced by the restoration of normal survival rates in strains supplemented with a plasmid carrying the *hisC* gene, in contrast to strains with the empty plasmid vector (Fig. 6B). In summary, these findings demonstrate that the metabolic pathways of glutamate and histidine are critical factors in antibiotic persistence and bacterial survival inside macrophages, respectively, in *A. baumannii*.

## Discussion

Bacteria can synthesize particular metabolites when faced with changes in their environment. In order to survive and proliferate during infection, pathogenic bacteria must adapt to the stresses present in their environment by modulating their metabolic pathways. In this study, we investigated that *A. baumannii*, a bacterium known for antibiotic resistance infections, is able to produce specific derivatives metabolites of amino acids and fatty acids. We determined that bacteria regulate specific metabolic pathways in a species-dependent manner (Figs. 1, 2 and 3). And then, comparative genomic analysis revealed that *A. baumannii* possesses specific genes or proteins in its metabolic pathways (Fig. 4 and Table S3). Moreover, we found that glutamate metabolism is essential for *A. baumannii* to promote antibiotic persistence (Fig. 7). Additionally, the enzyme HisC, which is involved in histidine biosynthesis, hinders the survival of *A. baumannii* inside macrophages (Fig. 7). These findings indicate that the metabolism of glutamate and histidine contributes to regulating bacterial physiology, including intramacrophage survival and antibiotic persistence, and could potentially serve as a novel target for therapeutic interventions against antibiotic resistance bacterium *A. baumannii*.

*A. baumannii* enhances antibiotic persistence through glutamate metabolism (Fig. 5), a stress response to harsh conditions such as antibiotic treatment, causing bacteria to become a dormant population [11]. The enzyme glutamate dehydrogenase GdhA synthesizes glutamate from α-ketoglutarate, an enzyme in the TCA cycle. Since TCA cycle inactivation enhances bacterial persister formation [28], inactivating *gdhA* leads to α-ketoglutarate accumulation, reducing persister formation by increasing TCA cycle activity in *A. baumannii* (Fig. 5) and uropathogenic *E. coli* [29]. Furthermore, the enzymes MurI1, AspB, and RacD use L-glutamate to generate D-glutamate and D-aspartate, essential for bacterial cell wall construction [30, 31] that leads to increase bacteria survival under stress conditions. D-form amino acids are also required for stress response via RpoS activation [32], potentially promoting antibiotic persistence. Consequently, *murI1*, *aspB*, or *racD* mutant strains exhibit reduced antibiotic persistence and increased susceptibility to antibiotics compared to wild-type strains. Together, the glutamate metabolic pathway promotes antibiotic persistence to some antibiotics by reducing central carbon metabolism and increasing cell wall construction and stress response [29–32].

The histidine biosynthesis pathway is typically required for bacterial survival inside macrophages [33–35]. For instance, the *his* operon, including the *hisC* gene, was highly upregulated in *Salmonella* inside macrophages [35]. Surprisingly, in *A. baumannii*, we found that inactivation of *hisC* increases the bacterial survival rate by about 4-fold in macrophages at 6 h post-infection (Fig. 6B). Inactivation of genes involved in histidine biosynthetic pathway decreases the L-histidine amount [36]. Interestingly, inactivation of the *hisF* gene in the *his* operon decreases cellular ATP levels 15-fold without severely decreasing GTP, UTP, and CTP levels under histidine starvation conditions similar to those inside the phagosome [37]. Low intracellular ATP levels induce persister formation [38]. Therefore, inactivation of the *hisC* gene may reduce intracellular ATP levels, which leads to persister formation when bacteria invade the phagosome, where L-histidine is deficient, conferring increased *A. baumannii* survival during infections.

Antibiotic-resistant bacteria continue to pose a significant threat to the health of humans, animals, and plants [38]. Stress response mechanisms that regulate bacterial physiology are involved in antibiotic persistence, which leads to the evolution of antibiotic-resistant bacteria [39]. Although global metabolite analysis has been shown to be a powerful tool for understanding bacterial physiology, this approach has not been widely used to examine and compare specific metabolic pathways across different bacterial species. This study contributes to the understanding of bacterial metabolite patterns and species-specific metabolites using MS-based global metabolic profiling, connecting them to bacterial physiology, such as antibiotic persistence and bacterial survival inside innate immune cells. By analyzing metabolic pathways through metabolite profiling, promising targets for inhibiting antibiotic-resistant bacteria could be identified. This approach offers a valuable tool for understanding and combating the growing problem of antibiotic resistance.

## Materials and methods

### Bacterial growth conditions

*E. coli* K12, *E. coli* O157:H7, *P. aeruginosa* PAO1, and *A. baumannii* ATCC 17,978 [40–43] were used throughout this study (Supplementary Table 3). Bacteria were cultured in modified M9 minimal medium containing Na_2_HPO_4_ ∙7H_2_O (6.8 g), KH_2_PO_4_ (3 g), NaCl (0.5 g), NH_4_Cl (1 g), MgSO_4_ (2 mM), and CaCl_2_ (0.1 mM) with glucose (2 g/L) and casamino acid (0.4%) at 37 ℃ under vigorous aeration. Three samples of each bacterium were cultured to evaluate global metabolite profiling and analyzed with three-technical replications.

### Quenching of the cultures and extraction of metabolites

Culture samples (5 mL) were plunged rapidly into an equal volume of 60% aqueous methanol solution (–50 ℃). The quenched biomass was centrifuged for 10 min at 3,000 × *g* and − 10 ℃. The supernatant was removed rapidly and retained to assess the extra-metabolites. The pellets and supernatants were snap frozen in liquid nitrogen and stored at − 80 ℃ for further analysis.

The biomass pellets were resuspended in 500 µL of 100% methanol (–50 ℃), frozen in liquid nitrogen, and allowed to thaw at − 20 ℃. The freeze-thaw cycle was performed three times to permeabilize the cells, resulting in leakage of the metabolites from the cells. The suspensions were centrifuged at 14,000 × *g*, at − 10 ℃, for 5 min. The supernatants were retained and stored at -20 ℃. An additional aliquot (500 µL) of 100% methanol (–50 ℃) was added to the pellet. The above procedure was repeated, and the second aliquot of methanol was combined with the first. The sample was stored at − 80 ℃ for analysis of intra-metabolites. All 5 mL supernatants were freeze-dried for 24 h, and then they were resuspended in 1 mL of 50% methanol (–50 ℃). The supernatants were stored at − 80 ℃ until LC/MS experiment.

### UPLC/Q-TOF-MS measurement

We employed both positive and negative electrospray ionization (ESI) LC/MS techniques to analyze the intracellular and extracellular metabolomes, which we refer to as intra- and extra-metabolomes, respectively. Intra- and extra-metabolite samples were filtered through a 0.2 μm membrane filter prior to analysis. The injection volume of the sample was 5 µL. Liquid chromatography was performed on a Waters Acquity UPLC system using an Acquity UPLC BEH C_18_ columns column (1.8 μm, 2.1 × 100 mm; Waters). The column oven temperature was maintained at 40 ℃, and the autosampler temperature was maintained at 4 ℃. The mobile phase was A (0.1% formic acid in water with 5% acetonitrile) and B (0.1% formic acid in 95% acetonitrile). The gradient started with 5% B and was increased linearly to 95% B within 12 min for ESI-negative mode analysis and within 14 min for ESI-positive mode analysis. The flow rate was 400 µL/min. Each sample was analyzed in both positive and negative ionization modes, using a hybrid quadrupole time-of-flight (Q-TOF) instrument; a TripleTOF 5600 fitted with a DuoSpray ion source (AB Sciex). Column effluent was directed to the ESI source. The source voltage was set to 5.0 kV for positive ionization and 4.0 kV for negative ionization mode. The declustering potential was 80 V and source temperature was 550 ℃ for both modes. The curtain gas flow, nebulizer, and heater gas were set to 25, 45, and 55 arbitrary units.

The instrument was set to perform one time-of-flight (TOF) MS survey scan (150 ms) and 20 MS/MS scans (50 ms each), with a total duty cycle time of 1.2 s. The mass range of both scan types was 50–1000 m/z. Acquisition of MS/MS spectra was controlled by the information dependent acquisition(IDA) function of the Analyst TF software (AB Sciex, Concord, Canada) with application of the following parameters; dynamic background subtraction, charge monitoring to exclude multiply charged ions and isotopes, and dynamic exclusion of former target ions for 5 s. Rolling collision energy (CE) was set whereby the software calculated the CE value to be applied as a function of m/z. Mass accuracy was maintained by the use of an automated calibrant delivery system (AB Sciex) interfaced to the second inlet of the DuoSpray source. Calibrations were performed at the start of each workday or whenever ionization polarity was changed.

### Data processing and multivariate statistical analysis

We processed the data using MS data processing software (MZmine), which ultimately generated a peak list [44, 45]. The efficiencies of two the software packages for LC/MS data processing, MZmine, were reportedly compared and gave similar results [46]. Recently, the METLIN MS/MS database was tested with several chemicals using five different instrument platforms [47]. For the determination of significant metabolites from the peak list, we performed a multivariate statistical analysis using SIMCA-P (version 12; Umetrics, Umea, Sweden). Principal component analysis (PCA), an unsupervised pattern-recognition (PR) method, was used to examine intrinsic variation in the Supplementary Dataset, and Partial least squares-discriminant analysis (PLS-DA), a supervised PR method, was employed to maximize the separation between samples. The quality of the models was described by *R*^*2*^and *Q*^*2*^ values. *R*^*2*^ is defined as the proportion of variance in the data explained by the models and indicates goodness of fit, and *Q*^*2*^ is defined as the proportion of variance in the data predictable by the model and indicates predictability. The explanation rates of the proposed model for the X and Y matrices are represented by R_2_X and R_2_Y, respectively, while Q_2_ indicates the model’s predictive power [45]. Generally, R_2_Y and Q_2_ values closer to 1 suggest a more stable and reliable model [26, 45], thereby demonstrating the goodness of fit and cross-validation predictive ability for our PLS-DA model (Table S2). In addition, we performed permutation tests to test the validity of the PLS-DA models. Variables having VIP > 1.5 in PLS-DA models were sorted. Statistical significance of selected variables was evaluated with Kruskal–Wallis tests (nonparametric ANOVA) and Tukey test using ranks as post-hoc test. And the *p*-values from Kruskal-Wallis test were corrected by false discovery rate (FDR) correction.

### Comparative genomic analysis of *A. baumannii* with bacteria

Comparative genomic analysis was performed to select for genes that potentially contribute to differential metabolite profile of *A. baumannii* ATCC 17,978. We first searched for the KEGG pathway maps (https://www.genome.jp/kegg/pathway.html) that are associated with the metabolism of differentially abundant metabolites for each of the studied strains (KEGG pathway IDs of 00360, 00400, 00350, 00471, 00250, 00290, 00280, 00061, 00071, 01040, and 01212). We then listed up the EC numbers of enzymes present in *A. baumannii*’s genome and searched if they were present in the other three strains. When they are present, we collected the nucleotide/protein sequence of the genes/enzymes from KEGG. Lastly, blastn/blastp (https://blast.ncbi.nlm.nih.gov/) were performed with default options to compare the gene/protein sequence of *A. baumannii* with the others. If align results have less than 60% in blastn and 30% in blastp, they are indicated as “Cannot align”. Also, if blastn and blastp analysis could not find any genes or proteins, it is indicated as “No maching gene”.

### Construction of metabolite MS/MS networks

Metabolite networks were generated for variables with VIP values > 1.5 using Cytoscape (www.cytoscape.org). The MS/MS spectral data were simplified by forming consensus spectra whereby identical spectra exhibiting identical precursor ion m/z and fragmentation patterns were combined. The simplified MS/MS data were then used for generation of the metabolite networks. Vector similarities were calculated for every possible pair of spectra with a minimum of six matching fragment ions with similarities determined by a modified cosine calculation that took into account the relative intensities of the fragment ions as well as the precursor m/z differences between the paired spectra. Cosine threshold values were set to 0.2 for each node, whereby a cosine value of 1.0 indicates identical MS/MS spectra. These data were imported into the Cytoscape program to visualize the MS/MS networks. Cytoscape produces a visual representation of the metabolite network where each node (i.e., circle) represents a single consensus MS/MS spectrum for a given parent mass, with the thickness of an edge between connecting nodes being indicative of the similarity score for that spectral pair, with higher scoring matches resulting in thicker connecting edges, and when possible, closer distances. Depending on Cytoscape’s nondeterministic network rendering algorithms, the distance between nodes also depends on the direction and number of connections.

### Identification of significant metabolites

For the identification of metabolites, MS/MS fragmentation patterns of selected metabolite groups from the network were compared to metabolomics databases (METLIN, KEGG, and LIPID MAPS). We performed hierarchical cluster analysis (HCA) dendrogram grouping of metabolites based on significant differences in relative abundance with selected metabolites from the network. The MS/MS fragmentation patterns of selected metabolites were then compared with the metabolomics databases, METLIN, KEGG, and LIPID MAPS. For detailed structural investigation of metabolites identified by clustering in the molecular network and metabolomics databases, we used a MS/MS spectra and chemical structure prediction program (SWATH, AB Sciex). Subsequently, if the MS/MS fragmentation patterns of our identified metabolites existed in the METLIN database, MS/MS data matching figure of our metabolites were generated with the METLIN MS/MS database and METLIN scores were calculated between the METLIN database and our MS/MS spectra.

### Construction of bacterial mutant strains and plasmid

All strains, oligonucleotides and plasmid used for mutant construction are listed in Table S4 and S5. For inactivation of *hisC*, *quiA*, *mmsB* and *fabF*, mutant strains were constructed with markerless gene deletion method [43]. Approximately 1000 bp of up and down stream region of target genes were amplified and combined by overlap extension PCR. Amplicons were digested with XbaI and XhoI (New England Biolabs, England) for 1 h at 37℃, and ligated with pDM4 plasmid with Quick Ligation™ Kit (New England Biolabs, England). Then plasmid was transformed to *A. baumannii* using MicroPulser Electroporator (Bio-Rad, USA). After transformation, *A. baumannii* harboring plasmid were plated onto LB agar supplemented with 10% sucrose to generate the deletion mutant strain by allelic exchange. For inactivation of *gdhA*, *murI1*,* murI2*, *aspB* and *racD*, mutant strains were constructed with one-step disruption method [48]. Briefly, kanamycin resistance gene of pKD4 [49] was amplified using primers bearing 125 bp of homologous region flanking the target genes by PCR. PCR product was transformed to *A. baumannii* expressing the pAT02 plasmid by electroporation using MicroPulser Electroporator (Bio-Rad, USA). After transformation, bacteria were plate onto LB agar containing kanamycin (50 µg/ml) to select the mutant strain generated by homologous recombination. By PCR, all mutant strains were confirmed whether target gene was deleted or not. For *gdhA*::FRT strain, kanamycin resistance gene was removed by pAT03 plasmid to prevent the growth delay by antibiotic pressure.

For complement plasmid construction, *hisC* and *gdhA* genes were amplified with its own promoter region (200 bp of upstream region from start codon). Amplicons were digested with EcoR1 and SalI (New England Biolabs, England) for 1 h at 37℃, and ligated with pWH1266 plasmid [50] with Quick Ligation™ Kit (New England Biolabs, England). Finally, Ligation product was transformed into each mutant strain by electroporation using MicroPulser Electroporator.

### Antibiotic persistence assay

Bacterial overnight culture was incubated at fresh M9 media after dilution (1:100) at 37℃, 180 rpm until early stationary phase (OD_600nm_ = 0.8 ~ 1.0), then norfloxacin (100 µg/ml), colistin (10 µg/ml), gentamicin (2 µg/ml) and ciprofloxacin (50 µg/ml) were added to culture. To determine the survival fraction, samples were collected every 2 h and washed 3 times with PBS to remove the remaining antibiotics. Washed samples were serially diluted with PBS and plated onto LB plate. Survival fraction was calculated by counting the colony forming unit (CFU) after incubating for 16 h at 37℃. Statistical analyses were performed using GraphPad Prism software. unpaired Student’s t tests were performed on the wild type and the other combinations. *P < *0.05*, **P < *0.01*, ***P < *0.001*, ****P < *0.0001* and ns: no significant.

### Intramacrophage survival assay

This experiment was performed following method described before [51]. The murine-derived macrophage cell line, J774A.1, was cultured in DMEM (WELGENE, Korea) supplemented with 10% FBS (WELGENE, Korea) at 37℃ under 5% CO_2_. The 5 × 10^5^ cells were seed at 24-well cell culture plates (SPL Life Sciences, Korea) for 20 h to prepare confluent monolayers. *A. baumannii* strains were used for infection at a multiplicity of infection (MOI) of 100. The bacterial overnight culture was diluted into fresh LB broth and incubated at 37℃, 180 rpm until the stationary phase (OD_600nm_ = 1). Bacteria were suspended with prewarmed DMEM medium, and then added to the well plates. The plates were incubated at 37℃ under 5% CO_2_ after centrifugation at 700 × g for 10 min. Following a 2 h incubation, the wells were washed 2 times with pre-warmed DPBS (WELGENE, Korea) and incubated at 37℃ under 5% CO_2_ supplemented with 200 µg/ml of gentamicin for 1 h to remove the extracellular bacteria. After incubation, plates were incubated at 37℃ under 5% CO_2_ supplemented with 10 µg/ml of gentamicin for 6 h. Following incubation, wells were washed 2 times with pre-warmed DPBS and 1 ml of 0.1% Triton X-100 was added to the wells and incubated for 15 min to lysate the cells. Bacteria were collected and serially diluted with PBS, then spotted onto LB agar plate and incubated at 37℃ 16 h to determine the number of surviving bacteria. Bacterial input was normalized based on optical density (OD_600nm_) as there was no significant difference in colony forming unit (CFU) and OD_600nm_ between wild-type and mutant strains. Survival rate was calculated by dividing the number of bacteria at 6 h.p.i. with the number of uptake bacteria. Statistical analyses were performed using GraphPad Prism software. unpaired Student’s t tests were performed on the wild type and the other combinations. *P < *0.05*, **P < *0.01*, ***P < *0.001* and ns: no significant.

### Growth kinetics

Bacterial overnight culture was diluted (1:100) with fresh M9 media and 100 µl of dilution was added into the sterilized 96 well. To prevent evaporation of samples 100 µl of mineral oil was added at each well. The plate was incubated at 37℃ while shaking continuously in the microplate reader.

### Determination of Minimal Inhibitory Concentration (MIC)

MIC of wild type and mutant strains against norfloxacin, colistin, gentamicin and ciprofloxacin were determined using the broth microdilution protocols with 96-well plate [52].

## Electronic supplementary material

Below is the link to the electronic supplementary material.

Supplementary Material 1

## Data Availability

No datasets were generated or analysed during the current study.

## Abbreviations

A. baumannii: Acinetobacter baumannii
P. aeruginosa: Pseudomonas aeruginosa
E. coli.: Escherichia coli
FDR: False Discovery Rate
KEGG: Kyoto Encyclopedia of Genes and Genomes
BLAST: Basic Local Alignment Search Tool
WHO: World Health Organization
PLS-DA: Partial Least Squares Discriminant Analysis
HCA: Hierarchical Cluster Analysis
MIC: Minimum Inhibitory Concentration
TCA: Tricarboxylic acid
ATP: Adenosine Triphosphate
GTC: Guanosine Triphosphate
UTP: Uridine Triphosphate
CTP: Cytidine Triphosphate
