# Supplementary material for: Regulation of antibiotic persistence and pathogenesis in Acinetobacter baumannii by glutamate and histidine metabolic pathways
Source: BMC Microbiol. 2025 Feb 14;25:74. doi: 10.1186/s12866-024-03654-1 (PMC11829494; doi:10.1186/s12866-024-03654-1)
Supplement: Supplementary file 1 — Supplementary Material 1 [file 12866_2024_3654_MOESM1_ESM.docx]

**Supplementary information for**

**Regulation of Antibiotic Persistence and Pathogenesis in *Acinetobacter baumannii* by Glutamate and Histidine Metabolic Pathways**

Ho Seok Sim^a,#^, Yong-Kook Kwon^b,c,d#^, Hokyung Song^e,#^, Geum-Sook Hwang^b,f*^ and Jinki Yeom^a,b,g,h,*^

^a^ Department of Biomedical Science, College of Medicine, Seoul National University, Seoul, 03080, Republic of Korea

^b^ Integrated Metabolomics Research Group, Western Seoul Center, Korea Basic Science Institute, Seoul 03760, Republic of Korea

^c^ Division of Food Safety Risk Assessment, National Institute of Food and Drug Safety Evaluation, Cheongju 28159, Republic of Korea

^d^Graduate School of Analytical Science and Technology, Chungnam National University, Daejeon, 305-764, Republic of Korea

^e^ Department of Environmental Engineering, Chosun University, Gwangju, 61452, Republic of Korea

^f^ College of Pharmacy, Chung-Ang University, Seoul 06974, Republic of Korea

^g^ Department of Microbiology and Immunology, College of Medicine, Seoul National University, Seoul, 03080, Republic of Korea

^h^ Cancer Research Institute, Seoul National University, Seoul, 08826, Republic of Korea

^#^ These authors contributed equally to this work

^*^Corresponding Authors: Jinki Yeom, [jinki.yeom@snu.ac.kr](mailto:jinki.yeom@snu.ac.kr); Geum-Sook Hwang, [gshwang@kbsi.re.kr](mailto:gshwang@kbsi.re.kr)

**Table S1.** Description of bacterial strains used in this study

| **Strains** | **Relevant characteristics** | **Description** | **Source** |
| --- | --- | --- | --- |
| *Escherichia coli* | wild type (MG1655) | Gram-negative bacteria model strain | (40) |
| *Escherichia coli* O157:H7 | wild type (43894) | Enterohemorrhagic strain, Shiga toxin 1, 2 producing strain | (41) |
| *Pseudomonas aeruginosa* | wild type (PAO1) | Opportunistic human pathogen, Antibiotic resistance bacteria | (42) |
| *Acinetobacter baumannii* | wild type (ATCC 17978) | Opportunistic human pathogen, Antibiotic resistance bacteria | (43) |

**Table S2.** Separated bacterial strains and model parameters from PLS-DA score plots.

|  | **Intra-metabolome** | | **Extra-metabolome** | |
| --- | --- | --- | --- | --- |
|  | **Positive** | **Negative** | **Positive** | **Negative** |
| **Strains**  (*R*^2^X/*R*^2^Y/*Q*^2^) | ***P. aeruginosa***  ***A. baumannii***  (0.518/0.992/0.936) | ***P. aeruginosa***  ***A. baumannii***  (0.731/0.994/0.955) | ***All strains***  (0.538/0.989/0.972) | ***P. aeruginosa,***  ***A. baumannii***  (0.554/0.990/0.963) |

**Table S3.** Distinct genes responsible for the synthesis of specific metabolites in *A.* *baumannii* after comparative genomic analysis

| **Description** | **Gene** | **Pathway_ID** | **acb_ID.x** | **KEGG ID** | **BLASTN^*^** | | | **BLASTP^*^** | | |
| --- | --- | --- | --- | --- | --- | --- | --- | --- | --- | --- |
|  |  |  |  |  | ***E. coli* K-12** | ***E. coli* O157:H7** | ***P. aeruginosa*** | ***E. coli* K-12** | ***E. coli* O157:H7** | ***P. aeruginosa*** |
| Phenylalanine metabolism | Phenylacetaldehyde dehydrogenase | 00360 | A1S_1852 | K00146 | Cannot align | No matching gene | Cannot align | 46.9% | No matching gene | 43.5% |
|  | Histidinol−phosphate aminotransferase* |  | A1S_0688 | K00817 | Cannot align | Cannot align | Cannot align | 30.1% | 30.1% | 55.5% |
|  | Amidase |  | A1S_1865 | K01426 | No matching gene | No matching gene | Cannot align | No matching gene | No matching gene | 30.1% |
|  | Acyl−CoA thioesterase |  | A1S_1349 | K02614 | Cannot align | No matching gene | No matching gene | 31.9% | No matching gene | No matching gene |
|  | FAD-dependent oxidoreductase |  | A1S_0951 | K00529 | Cannot align | Cannot align | No matching gene | 31.8% | 30.8% | No matching gene |
| Phenylalanine, tyrosine and tryptophan biosynthesis | Tryptophan synthase alpha chain | 00400 | A1S_2870 | K01695 | Cannot align | Cannot align | Cannot align | 32.8% | 33.2% | 48.3% |
|  | Quinate dehydrogenase* |  | A1S_1880 | K05358 | No matching gene | No matching gene | No matching gene | No matching gene | No matching gene | No matching gene |
|  | Tryptophan synthase beta chain |  | A1S_1692 | K06001 | No matching gene | No matching gene | No matching gene | No matching gene | No matching gene | No matching gene |
|  | 3−Phosphoshikimate 1−carboxyvinyltransferase |  | A1S_2276 | K24018 | No matching gene | No matching gene | No matching gene | No matching gene | No matching gene | No matching gene |
|  | Chorismate mutase |  | A1S_2084 | K04093 | No matching gene | No matching gene | Cannot align | No matching gene | No matching gene | 29.2% |
| Tyrosine metabolism | Maleylacetoacetate isomerase | 00350 | A1S_3415 | K01800 | No matching gene | No matching gene | No matching gene | No matching gene | No matching gene | No matching gene |
|  | Aspartate aminotransferase* |  | A1S_2508 | K00812 | No matching gene | No matching gene | No matching gene | No matching gene | No matching gene | No matching gene |
|  | 5−Carboxymethyl−2−hydroxymuconate isomerase |  | A1S_2096 | K01826 | No matching gene | No matching gene | No matching gene | No matching gene | No matching gene | No matching gene |
|  | 4−Hydroxyphenylacetate 3−hydroxylase |  | A1S_1856 | K23470 | No matching gene | No matching gene | No matching gene | No matching gene | No matching gene | No matching gene |
| D-Glutamine and D-glutamate metabolism | Glutamate racemase I* | 00471 | A1S_0380 | K01776 | Cannot align | Cannot align | Cannot align | 37.5% | 37.5% | 43.3% |
|  | Glutamate racemase II* |  | A1S_3398 | K01776 | Cannot align | Cannot align | Cannot align | 34.9% | 34.9% | 39.5% |
| Alanine, aspartate and glutamate metabolism | Glutamate dehydrogenase (NAD(P)+)* | 00250 | A1S_3134 | K00261 | No matching gene | No matching gene | No matching gene | No matching gene | No matching gene | No matching gene |
|  | Aspartate racemase* |  | A1S_2202 | K01779 | No matching gene | No matching gene | No matching gene | No matching gene | No matching gene | No matching gene |
| Valine, leucine and isoleucine degradation | Carbamoyl-phosphate synthase | 00280 | A1S_1270 | K11263 | No matching gene | No matching gene | No matching gene | No matching gene | No matching gene | No matching gene |
|  | 3−Hydroxyisobutyrate dehydrogenase* |  | A1S_0572 | K00020 | No matching gene | No matching gene | Cannot align | No matching gene | No matching gene | 49.6% |
| Phenylpropanoid biosynthesis. | 4-Hydroxyphenylpyruvate dioxygenase | no pathway | A1S_3418 | K00457 | No matching gene | No matching gene | 70.3% | No matching gene | No matching gene | 50.0% |
|  | Pyrroline-5-carboxylate reductase |  | A1S_0610 | K00286 | No matching gene | No matching gene | No matching gene | No matching gene | No matching gene | No matching gene |
| Phenolic fatty acid Lysine derivatives | Lysine N6-hydroxylase | no pathway | A1S_2384 | K03897 | No matching gene | No matching gene | No matching gene | No matching gene | No matching gene | No matching gene |
|  | Lysine-specific permease |  | A1S_2838 | K11733 | No matching gene | No matching gene | No matching gene | No matching gene | No matching gene | No matching gene |
| Fatty acid metabolism | 3-Oxoacyl-[acyl-carrier-protein] synthase II* | 01212 | A1S_0525 | K09458 | 90.9% | 64.2% | Cannot align | 38.8% | 61.0% | 35.5% |
|  | Long-chain acyl-CoA synthetase |  | A1S_1815 | K01897 | 80.3% | 81.7% | Cannot align | 44.3% | 44.6% | 44.1% |

**^*^**Each score indicates similarity analyzed with BLAST. No matching gene indicates that no genes are found with BLAST, and cannot align indicates that similarity score were less than 60% for BLASTn and less than 30% for BLASTp.

**Table S4.** Bacterial strains and plasmids used for genetic mutation in this study

| **Strains** | **Relevant characteristics** | **Source** |
| --- | --- | --- |
| ***Escherichia coli*** | | |
| DH5a | Host strain used for generation and propagation of plasmid constructs | Life Technologies |
| ***Acinetobacter baumannii*** | | |
| HS61 | Wild type (ATCC 17978) | (43) |
| HS81 | Δ*fabF* | This study |
| HS171 | Δ*mmsB* | This study |
| HS173 | Δ*quiA* | This study |
| HS175 | Δ*hisC* | This study |
| HS237 | Δ*murI2*::Kan | This study |
| HS239 | Δ*gdhA*::Kan | This study |
| HS241 | Δ*murI1*::Kan | This study |
| HS243 | Δ*aspB*::Kan | This study |
| HS245 | Δ*racD*::Kan | This study |
| HS261 | Δ*gdhA*::FRT | This study |
| **Plasmids** | | |
| pDM4 | rep_R6Kg_ Km^R^ SacB | (43) |
| pAT02 | rep_RSF1010_ Amp^R^ Rec_Ab_ stytem | (48) |
| pAT03 | rep_RSF1010_ Amp^R^ FLP recombinase | (48) |
| pKD4 | rep_R6Kg_ Amp^R^ FRT Kan^R^ FRT | (49) |
| pWH1266 | rep_pBR322_ rep_pWH1277_ Amp^R^ Tet^R^ | (50) |
| pWH1266::*hisC* | rep_pBR322_ rep_pWH1277_ Amp^R^ *hisC* | This study |
| pWH1266::*gdhA* | rep_pBR322_ rep_pWH1277_ Amp^R^ *gdhA* | This study |

**Table S5.** Primers used for strain, plasmid constructions in this study

| **Name** | **Purpose** | **Sequence (from 5' to 3')** |
| --- | --- | --- |
| *fabF*_UP_F | Amplification approximately 1 kb of up stream gene of *fabF* | TACCCGCATGCAAGATCTATGCCTTAGGTTCACGATTGATC |
| *fabF*_UP_R | Amplification approximately 1 kbp of up stream gene of *fabF* | GTTGTGTTCCGTAACGAATACCGCTTTTAGCAG |
| *fabF*_DW_F | Amplification approximately 1 kb of down stream gene of *fabF* | TATTCGTTACGGAACACAACAATGTTAAATAAAAAACTTTTATTTGTAG |
| *fabF*_DW_R | Amplification approximately 1 kb of down stream gene of *fabF* | CTTATCGATACCGTCGACCCAAGCTCATGCGAACATAATCCG |
| *mmsB*_UP_F | Amplification approximately 1 kb of stream gene of *mmsB* | TACCCGCATGCAAGATCTATCTAGAAACTAAAGCGTCATGGACGAC |
| *mmsB*_UP_R | Amplification approximately 1 kb of up stream gene of *mmsB* | GAGCTTTATTTAAACGGAGTTTGTGTATGGGTC |
| *mmsB*_DW_F | Amplification approximately 1 kb of down stream gene of *mmsB* | ACTCCGTTTAAATAAAGCTCTTATCTATATTGGCGTGG |
| *mmsB*_DW_R | Amplification approximately 1 kb of down stream gene of *mmsB* | CTTATCGATACCGTCGACCCTCGAGGACGATGGCTTCATTGAAGAAG |
| *quiA*_UP_F | Amplification approximately 1 kb of up stream gene of *quiA* | TACCCGCATGCAAGATCTATCTAGAAATTTTGTTTCTGTACCATTTGGTTGAATC |
| *quiA*_UP_R | Amplification approximately 1 kb of up stream gene of *quiA* | TTTTTATATCTGAAAGAGGGCAAAAGGTTAAATTTTC |
| *quiA*_DW_F | Amplification approximately 1 kb of down stream gene of *quiA* | CCCTCTTTCAGATATAAAAAATACCAGTAGCCAATAAAGCTAAAC |
| *quiA*_DW_R | Amplification approximately 1 kb of down stream gene of *quiA* | CTTATCGATACCGTCGACCCTCGAGAATAGTTCTCAATGTGAGTTTCAGAACTTATTG |
| *hisC*_UP_F | Amplification approximately 1 kb of up stream gene of *hisC* | TACCCGCATGCAAGATCTATCTAGATGTACTTATGAATGCGGTACCAG |
| *hisC*_UP_R | Amplification approximately 1 kb of up stream gene of *hisC* | AGAATAAGTAATACGGCTCTAATTCACGAACC |
| *hisC*_DW_F | Amplification approximately 1 kb of down stream gene of *hisC* | AGAGCCGTATTACTTATTCTGCTGTGCTGTTCATC |
| *hisC*_DW_R | Amplification approximately 1 kb of down stream gene of *hisC* | CTTATCGATACCGTCGACCCTCGAGACAACCGTCTTTATTTATAGGTTCCTATTATTC |
| *murI2*_DelRec_F | Amplification of *murI2::Kan* to inactivate *murI2* gene | AAGTCGTGCTCAACGACTTGGCAACCATGAAAGAATTTTTCTAAATATATGAAATACAGGATAAAGCATGGTGTAGGCTGGAGCTGCTTC |
| *murI2*_DelRec_R | Amplification of *murI2::Kan* to inactivate *murI2* gene | GAAGCCCCAAAAATCTATGTCAGTGATGGGTGGACTGATTTTTATGGGGCTAGGCGTTTTTCTTGGCTTTATATGAATATCCTCCTTAGT |
| *gdhA*_DelRec_F | Amplification of *gdhA::Kan* to inactivate *gdhA* gene | ACAAATAACCCTATAACATCCTTTATATTTGCTCGACCCACCTTTCCTAAAAGGTAGTTTCCTCAGATTAGTGTAGGCTGGAGCTGCTTC |
| *gdhA*_DelRec_R | Amplification of *gdhA::Kan* to inactivate *gdhA* gene | ATTTAGCTGATTATATCGATACATTAAAACGTCCTAAGCGTGCGCTTATTGTGGACGTGCCAATTGTAATGTATGAATATCCTCCTTAGT |
| *murI1*_DelRec_F | Amplification of *murI1::Kan* to inactivate *murI1* gene | CTTGCAATTAGGCACTTGAGGTTTAAGCTTTATAGAGTGAAATTTTCCGACACTCTCTAGTACTGCCATGGTGTAGGCTGGAGCTGCTTC |
| *murI1*_DelRec_R | Amplification of *murI1::Kan* to inactivate *murI1* gene | TATGAACCGCCCCAAATAAGAAAAATAAATATAAAAAAACGCTTTCTTCTGAATGCCTACAACAGAGCTCATATGAATATCCTCCTTAGT |
| *aspB*_DelRec_F | Amplification of *aspB::Kan* to inactivate *aspB* gene | TATCAAACCGTCCCCTACACTTGCTGTAACTAATAAAGCTGCTGAGCTTAAAGCTGCTGGCAAGAACGTGGTGTAGGCTGGAGCTGCTTC |
| *aspB*_DelRec_R | Amplification of *aspB::Kan* to inactivate *aspB* gene | ATAAAAATCAAACTAATAAAAAAGGAAGTTTTAAATAAAAACTTCCTTTTTAGATTTATAACTAATAATTATATGAATATCCTCCTTAGT |
| *racD*_DelRec_F | Amplification of *racD::Kan* to inactivate *racD* gene | GCTGGCTTTCAACAAATTGGGCTGTGATTCGTTCGTATTTCAGAATAAATTAAAAGTTGATGAGTATTTAGTGTAGGCTGGAGCTGCTTC |
| *racD*_DelRec_R | Amplification of *racD::Kan* to inactivate *racD* gene | AAACAATCGGTTTACTCGGCGGTATGAGCTGGGAATCTACATCACTTTACTATCAACAAATTAACAGAATGTATGAATATCCTCCTTAGT |
| *fabF*_Veri_F | To verify the *fabF* gene deletion | GATGAAGTCAAAGAACATGC |
| *fabF*_Veri_R | To verify the *fabF* gene deletion | CCGACTTACCAAAACCATTG |
| *mmsB*_Veri_F | To verify the *mmsB* gene deletion | CAGTACGCCATAAGCTTTCT |
| *mmsB*_Veri_R | To verify the *mmsB* gene deletion | TCATTCAAATTTTAACGCAATGGAC |
| *quiA*_Veri_F | To verify the *quiA* gene deletion | GCCTAATTGTTCAGGTAATTTTTCC |
| *quiA*_Veri_R | To verify the *quiA* gene deletion | TTATCCCAAGCATGGGT |
| *hisC*_Veri_F | To verify the *hisC* gene deletion | GATGGTTTAAATCAAGAGATTCGTG |
| *hisC*_Veri_R | To verify the *hisC* gene deletion | AGTTAACTGGACTTGTATATCTTCA |
| *murI2*_Veri_F | To verify the *murI2* gene deletion | GTAAAAACCAAGCAATTTATGTCG |
| *murI2*_Veri_R | To verify the *murI2* gene deletion | CATCATTAGATTTTCCAGTTTGGT |
| *gdhA_*Veri_F | To verify the *gdhA* gene deletion | ACTGGAACCATCCAGT |
| *gdhA*_Veri_R | To verify the *gdhA* gene deletion | CAGATTTATCACATCTGAGTCTG |
| *murI1*_Veri_F | To verify the *murI1* gene deletion | CTTATGTCCATGTTCGATTTG |
| *murI1*_Veri_R | To verify the *murI1* gene deletion | GTTTGGGAAAGAACCATAC |
| *aspB*_Veri_F | To verify the *aspB* gene deletion | CATCTAAAAATCCCCACATC |
| *aspB*_Veri_R | To verify the *aspB* gene deletion | TAATCTTTGCCAAGCTGTC |
| *racD*_Veri_F | To verify the *racD* gene deletion | ATTACCATGCTGGTGGTT |
| *racD*_Veri_R | To verify the *racD* gene deletion | GCGGTCTGTTTCTAACATAAAG |
| *hisC*_Comp_F | Amplification of *hisC* gene with own promoter for complementation | TTCAAGAATTCCTGATGTACTTGCT |
| *hisC*_Comp_R | Amplification of *hisC* gene with own promoter for complementation | CATCGGTCGACTTAAAGAATATCTT |
| *gdhA*_Comp_F | Amplification of *gdhA* gene with own promoter for complementation | TTCAAGAATTCCAGATTTATCACAT |
| *gdhA* _Comp_R | Amplification of *gdhA* gene with own promoter for complementation | CATCGGTCGACTTAGCCTGGGAAAA |
| pWH1266_Veri_F | To verify the complementation with pWH1266 plasmid | ACATTTCCCCGAAAAGTG |
| pWH1266_Veri_R | To verify the complementation with pWH1266 plasmid | CGAGTTGCATGATAAAGAAGAC |

**Table S6.** Minimal inhibitory concentration (MIC) of wild type and mutant strains.

|  | **Minimal inhibitory concentration (μg/ml)** | | | | |
| --- | --- | --- | --- | --- | --- |
| **Antibiotics** | **Wild type** | **Δ*gdhA*** | **Δ*murI1*** | **Δ*aspB*** | **Δ*racD*** |
| Norfloxacin | 16 | 16 | 16 | 16 | 16 |
| Colistin | 1 | 1 | ND | ND | ND |
| Gentamicin | 1 | 1 | ND | ND | ND |
| Ciprofloxacin | 1 | 1 | ND | ND | ND |

**Figure S1. Permutation test of PLS-DA models.** Permutation tests of all PLS-DA models were performed for Gram-negative bacteria using 100 permutations. Permutation plots for the PLS-DA models showing R_2_ (green) and Q_2_ (blue) values. Intra(+), intra-metabolite assessed by ESI-positive mode (A); Intra(-), intra-metabolite assessed by ESI-negative mode (B); Extra(+), extra-metabolite assessed by ESI-positive mode (C); Extra(-), extra-metabolite assessed by ESI-negative mode (D).

**Figure S2. Partial MS/MS network in Gram-negative bacteria analysis and annotation of MS/MS spectra for reported compounds about Phe / Glu derivatives metabolites.** A. Partial annotated MS/MS network of Gram-negative bacteria analysis. B. Annotation of the MS/MS for 3,4-Pyridindiamin in Phe and Glu

derivatives. The MS/MS spectra for compound and the red arrow indicated precursor ion. C. Annotation of the MS/MS for Glutathione in Phe and Glu

derivatives. The MS/MS spectra for compound and the red arrow indicated precursor ion. Automated MS/MS data matching with METLIN MS/MS database was performed and METLIN score was also calculated between METLIN database and our MS/MS spectra (bottom right figure). D. Annotation of the MS/MS for Methyl 3-[(3-nitrophenyl)amino]-2-butenoate in Phe and Glu derivatives. The MS/MS spectra for compound and the red arrow indicated precursor ion. E. Annotation of the MS/MS for N-(4-nitrobenzoyl)glutamate in Phe and Glu derivatives. The MS/MS spectra for compound and the red arrow indicated precursor ion.

**Figure S3. Partial MS/MS network in Gram-negative bacteria analysis and annotation of MS/MS spectra for reported compounds about Phe / Leu derivatives metabolites.** A. Partial annotated MS/MS network of Gram-negative bacteria analysis. B. Annotation of the MS/MS for N1-Acetylspermidine in Pro and Leu derivatives. The MS/MS spectra for compound and the red arrow indicated precursor ion. C. Annotation of the MS/MS for Leu-leu in Pro and Leu derivatives. The MS/MS spectra for compound and the red arrow indicated precursor ion. D. Annotation of the MS/MS for Thr-leu in Pro and Leu derivatives. The MS/MS spectra for compound and the red arrow indicated precursor ion.

**Figure S4. Partial MS/MS network in Gram-negative bacteria analysis and annotation of MS/MS spectra for reported compounds about lipids metabolites.** A. Partial annotated MS/MS network of Gram-negative bacteria analysis. B. Annotation of the MS/MS for 3-O-alpha-L-rhamnopyranosyl-3 hydroxydecanoyl-3-hydroxydecanoic acid in Rhamnolipid. The MS/MS spectra for compound and the red arrow indicated precursor ion.

**Figure S5. Partial MS/MS network in Gram-negative bacteria analysis and annotation of MS/MS spectra for reported compounds about quinolones metabolites.** A. Partial annotated MS/MS network of Gram-negative bacteria analysis. B. Partial annotated Heat map and HCA analysis depicting compounds of the upper MS/MS network. C-G. Annotation of the MS/MS for 2-Heptyl-1-hydroxy-4(1H)-quinolinone (C), 2-[(1-Butyl-2-naphthyl)oxy]-N,N-dimethylethanamine (D), 3-Hydroxymorphinan (E), 1,2-Bis(3-methoxyphenyl)ethanamine (F) and 1-[(2,4-Dimethyl-1-piperidinyl)methyl]-2-naphthol (G). The MS/MS spectra for compound and the red arrow indicated precursor ion.

**Figure S6. Partial MS/MS network in Gram-negative bacteria analysis and annotation of MS/MS spectra for reported compounds about pyridine derivatives and vitamin derivatives metabolites.** A. Partial annotated MS/MS network of Gram-negative bacteria analysis. B. Partial annotated Heat map and HCA analysis depicting compounds of the upper MS/MS network. C-F. Annotation of the MS/MS for 14-Hydroxy-5-tetradecenoic acid (C), Dihydropteridine (D), 5-Heptyl-5-methyl-1,3-oxazolidine-2,4-dione (E), and 5 Butyryl-L-carnitine (F). The MS/MS spectra for compound and the red arrow indicated precursor ion.

**Figure S7. Partial MS/MS network in Gram-negative bacteria analysis and annotation of MS/MS spectra for reported compounds about pyridine and Glu derivatives metabolites.** A. Partial annotated MS/MS network of Gram-negative bacteria analysis. B. Partial annotated Heat map and HCA analysis depicting compounds of the upper MS/MS network. C-D. Annotation of the MS/MS for N-Acetyl-L-glutamic acid (C) and Glutamylphenylalanine (D). The MS/MS spectra for compound and the red arrow indicated precursor ion.

**
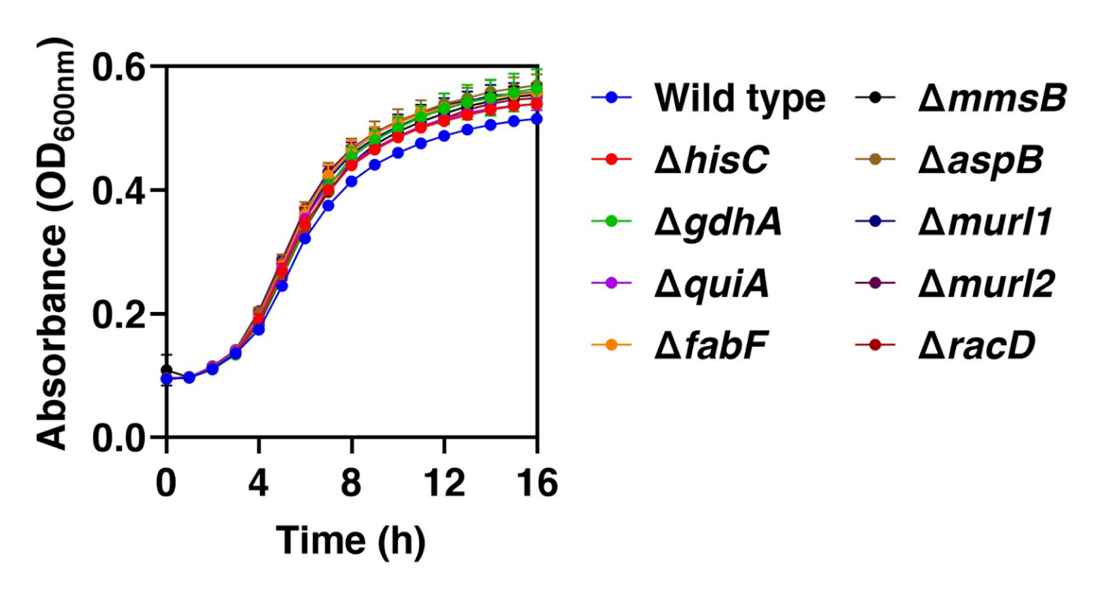
**

**Figure S8. Growth kinetics of *A. baumannii* strains.** Growth kinetics was performed with wild type and mutant strains in the M9 media. Bacteria were incubated at 37 °C for 16 hours.

**
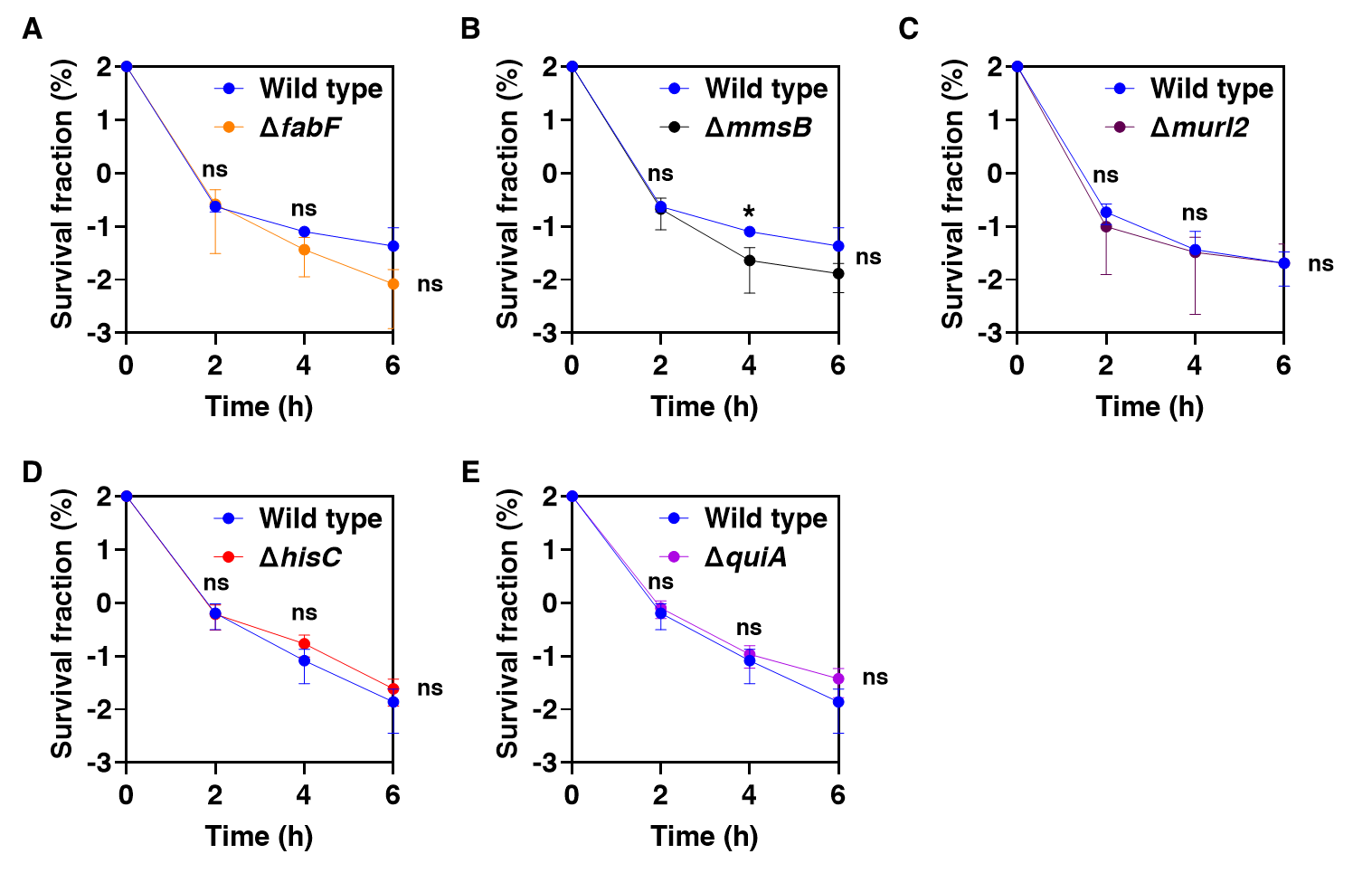
**

**Figure S9. Time killing curve of *A. baumannii* under norfloxacin treatment.** Killing curves was generated with wild type and mutant strains under norfloxacin (100 μg/ml). Survival fraction was calculated with bacterial number before antibiotic treatment. Statistical analyses were performed using GraphPad Prism software. Unpaired Student’s t tests were performed on the wild-type sample and the other combinations *(*p < 0.05* and ns: no significant).


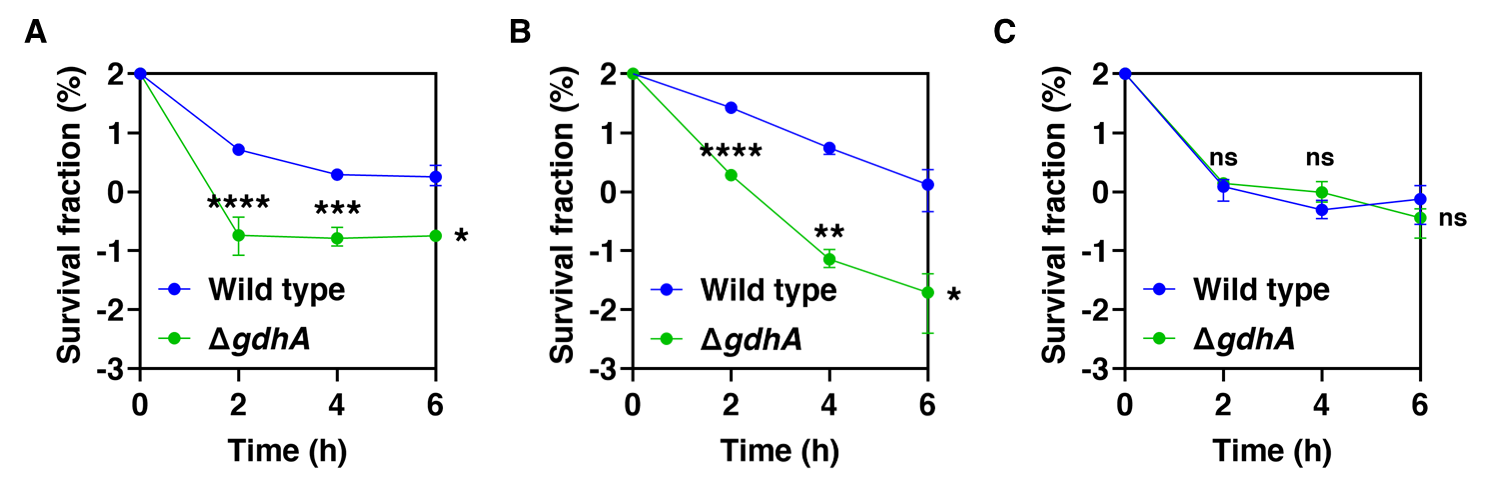


**Figure S10. Time killing curve of *A. baumannii* under antibiotics treatment.** Killing curves was generated with wild type and mutant strains under (A) colistin (10 μg/ml), (B) ciprofloxacin (50 μg/ml) and (C) gentamicin (2 μg/ml). Survival fraction was calculated with bacterial number before antibiotic treatment. Statistical analyses were performed using GraphPad Prism software. Unpaired Student’s t tests were performed on the wild-type sample and the other combinations *(*p < 0.05*, ***p < 0.01*, ****p < 0.001*, ****p < 0.001* and ns: no significant).
